# Does ‘You Are What You Eat’ Apply to Mangrove Grapsid Crabs?

**DOI:** 10.1371/journal.pone.0089074

**Published:** 2014-02-13

**Authors:** Thi Hong Hanh Bui, Shing Yip Lee

**Affiliations:** Australian Rivers Institute and School of Environment, Griffith University Gold Coast Campus, Southport, Qld, Australia; North Carolina State University, United States of America

## Abstract

In tropical mangroves, brachyuran crabs have been observed to consume high percentages of leaf litter production. However, questions concerning their ability to assimilate this low-quality food remain, as stable isotope analysis of C and N does not seem to support assimilation. Individuals of the common eastern Australian mangrove grapsid *Parasesarma erythodactyla* feeding on a mangrove leaf litter or mangrove+microphytobenthos diet developed a significantly higher hepatosomatic index than those with access to only sediment. Lipid biomarker analysis and feeding experiments using ^13^C and ^15^N-enriched mangrove leaf litter confirmed rapid assimilation of mangrove C and N by *P. erythodactyla*. Eight-week feeding experiments utilizing three food types (mangrove leaf litter, microphytobenthos and prawn muscle) established different food-specific trophic discrimination values (Δδ^13^C and Δδ^15^N) that are significantly different from those commonly applied to mixing model calculations. The mean Δδ^13^C_(crab-mangrove)_ of +5.45‰ was close to the mean and median literature values for grapsid-mangrove pairs in 29 past studies (+5.2±1.8‰ and +5.6‰, respectively), suggesting that this large discrimination may generally be characteristic of detritivorous grapsid crabs. Solutions from the IsoConc mixing model using our determined trophic discrimination values suggest significantly higher and dominant contributions of mangrove C to the diet than those based on the global mean trophic discrimination values. Our results reaffirm the physiological capacity for and important mediating role of grapsid crabs in processing low-quality mangrove C in tropical estuaries, and caution against the use of global trophic discrimination values in stable isotope analysis of food-web data, especially those involving detritivores. While recent studies have questioned the trophic significance of mangrove detritus in coastal food chains, the contribution of this productive carbon source needs to be re-assessed in the light of these data.

## Introduction

Despite their limited global areal extent of *ca.* 1.52×10^5^ km^2^ [1]–[2], tropical mangrove forests are amongst the most productive ecosystems on earth [3]–[4]. Early paradigms on estuarine carbon dynamics emphasized the ‘outwelling’ role of mangroves and saltmarshes in subsidising nearshore consumers with organic matter [5]–[7], but the spatial extent and the trophic significance of exported vascular plant detritus in nearshore food webs seem to be overstated [4], [8]. The generally low nutritive value (e.g. high C/N ratio) and recalcitrant nature (e.g. high structural carbon content) of mangrove and saltmarsh plant detritus has prompted recent suggestions that this production may be consumed or mineralized minimally (eg. [9]–[10]), thus promoting storage. Other reports argued that even when this detritus is consumed, it may lead to trophic ‘cul-de-sacs’ [11].

The notion that mangrove detritus contributes little to nearshore consumer food chains is apparently supported by the stable isotope tracer analysis data. Numerous studies, using stable isotope analysis of carbon, nitrogen and occasionally sulphur, covering a range of mangrove-dominated systems, have yielded consumer signatures deemed too distant (e.g. >+5‰ for δ^13^C) to directly relate consumer biomass to assimilated mangrove detritus material (e.g. [12]–[14]; review in [15]). Alternatively, more ^13^C-enriched producers such as the microphytobenthos (MPB), seagrass and phytoplankton have been suggested as the primary C sources. This notion also corroborates with the paradigm that algal C is more easily utilizable by animals compared to mangrove leaf litter (with a low N content but high concentration of secondary metabolites).

While the trophic dependence of nearshore consumers on mangrove detritus is debatable, there is much direct evidence of consumption of this relatively poor food source by grapsid crabs (Brachyura: Grapsidae) throughout the Indo-west-Pacific (IWP) [16]–[19], where these crabs are diverse and abundant [20]. Gut content analysis revealed that mangrove leaf litter was the dominant food item of the mangrove grapsids, though evidence of animal tissue and sediment consumption was also recorded [21]–[23]. With high consumption rates, significant percentages (>50%) of mangrove leaf litter production were retained and processed via this pathway, potentially ‘short-circuiting’ the food chain [24]. Initial processing by grapsid crabs may physically and biologically facilitate subsequent utilization of mangrove detritus by other consumers in nearshore food chains. The crabs act as shredders of fresh or aged leaf litter, greatly reducing the size but increasing the surface area to volume ratio of processed fragments, thus enhancing microbial colonisation and physical leaching of feeding deterrents [25]–[26]. The crabs therefore act as an efficient initial processor for low-quality mangrove leaf litter material, mediating its eventual utilization by other estuarine consumers [20]. This role is, however, dependent on the crabs’ ability to effectively digest and assimilate the low-quality mangrove leaf litter diet.

Recently, the nutritional dependence of mangrove grapsids on mangrove leaf litter, and thus implicitly their role in acting as initial processors of mangrove organic production, has been questioned. Firstly, the physiological mechanism that allows profitable utilization of this poor food source is unknown. Secondly, consumption of mangrove leaf litter, often fresh and without microbial enrichment, by the grapsids is considered trophically non-viable and there needs to be substantial additional nutrient sources particularly for N [27]–[28]. Finally, stable isotope analysis of the leaf litter-crab link apparently fails to produce data that support the dominant contribution of mangrove C in the grapsids’ diet. For example, recently Mazumder and Saintilan [9] claimed that mangrove (*Avicennia marina*) leaf litter could not be an important food source for the temperate grapsid crabs *Helograpsus haswellianus* and *Parasesarma erythodactyla* in Australia, as the δ^13^C of the crabs were too high to indicate substantial utilization of this food item. There is a consistent large difference between the δ^13^C of mangrove leaf litter and those of consumers potentially benefitting from the mangrove detritus-based food chain, e.g. [15], [27], [29]. It should be noted, however, that the interpretation of stable isotope data (particularly δ^13^C and δ^15^N) are based on a few assumptions, such as a constant trophic discrimination factor between consumer and food. In most analyses, values of ∼ +1‰ and ∼+3‰ have been used for generating solutions from mixing models relating mangrove detritus and crab biomass δ^13^C and δ^15^N values, respectively. The applicability of these values generated from a wide range of consumer-food combinations to explaining specific trophic paths such as the grapsid crab-mangrove detritus link has not been tested. Significant deviations from these assumed values would have strong impacts on the interpretation of the stable isotope data, and thus the importance of the trophic links concerned.

In order to reconcile the observation that mangrove grapsids do consume large quantities of mangrove leaf litter, and the apparently contradictory stable isotope data related to this important link in mangrove food chains, we conducted a series of feeding experiments utilizing naturally abundant and isotopically enriched substrates to (1) investigate the ability of the mangrove grapsid *P. erythodactyla*, a common grapsid crab of the subtropical mangroves in eastern Australia, to directly utilize leaf litter of *Avicennia marina*; (2) empirically determine the trophic discrimination values of its potential food sources, namely mangrove leaves, MPB, and animal tissue; (3) estimate and compare diet compositions predicted from IsoConc mixing modelling based on the commonly assumed and our determined trophic discrimination values, and (4) discuss the implication of our results for the evaluation of the mangrove detritus food chain.

## Methods

No ethics approval is required for this study according to current Australian law. The species used (*Parasesarma erythodactyla*) is not an endangered or a protected species. No specific permission is required for collection of grapsid crabs from the study location.

### Collection of Crabs, Mangrove Leaf Litter and Sediment

Intermoult male individuals of *P. erythodactyla* of carapace width ranging from 11 to 20 mm were collected from an *A. marina*-dominated intertidal mangrove forest at Tallebudgera Creek, southeast Queensland, Australia. Crabs that were analyzed later as ‘field’ samples (hereafter referred as T0 sampling event) were rinsed with distilled water and frozen immediately upon arrival to the laboratory while those for the laboratory experiments were transferred to individual growth compartments (l×w×h* = *13×7×4 cm, each containing 50 ml of sea water) and starved for two days for acclimation to laboratory condition and gut evacuation.

Freshly senescent mangrove leaves (yellow leaves that were easily detached from the trees), MPB, and sediment were collected from the same sites where the crabs were caught. Leaves were soaked in seawater for 24 h to remove feeding deterrents, e.g. tannin, before being offered to crabs. Sediments were collected by scraping the top 1 cm surface sediment, homogenized with a shovel before adding into experimental tanks as an organic substrate in experiment 1 or used for MBP isolation.

#### Extraction of MPB from sediment

MPB was isolated from sediment by density gradient centrifugation in colloidal silica. Sediment was spread to ca. 3 cm depth in plastic trays (45×30×5 cm), which were exposed to white fluorescent light for 16 h to mediate vertical migration of MBP to the surface of the sediment. The top ca. 0.5 cm sediment was then scrapped, suspended in seawater, and then sieved through a 63 µm to remove large detritus and nematodes. The filtrate was centrifuged at 4400 rpm for 5 min. Supernatant was poured off. Pellets were resuspended in left over supernatant, divided into 5 ml aliquots in individual centrifuge tubes, mixed with 40 ml of 30% Ludox colloidal silica (Sigma), and centrifuged at 4400 rpm for 5 min. The distinct layer of MPB, which was confirmed by microscopic examination, was collected, and washed with distilled water to remove Ludox before collected on pre-combusted glass filters. Each filter was loaded with the MPB suspension until it was clogged to maximize amount of MPB provided as a food source to the crabs in experiment 3. MPB containing filters were stored at −20°C until used. Aliquots of MPB were dried at 60°C for stable isotope analysis.

#### Preparation of ^13^C and ^15^N enriched mangrove leaves

Thirty *Avicennia* seedlings each with 4–6 leaves, were planted in two glass chambers (h×l×w = 40×50×30 cm) containing 10 cm deep sediment. Seedlings were grown at 24°C, under lighting from fluorescent tubes. The seedlings were labelled with ^13^C and ^15^N using methods modified from Bromand *et al.* [30] and Unsicker *et al.* [31]. Growth chambers were left open for two days, allowing water to evaporate from the top sediment before 1 ml of 61.2 mM ^15^NH_4_Cl (99 atom% ^15^N, Cambridge Isotope Laboratories) was injected at each of the 15 injection points evenly distributed in each chamber. Injection was done at the depth of 2 cm from sediment surface using a 1 ml syringe. The top sediment was then re-wetted with distilled water. For ^13^C labelling, a bottle containing 25 ml of 1 M NaH^13^CO_3_ (99 atom% ^13^C, Cambridge Isotope Laboratories) was placed in each chamber before it was tightly sealed. One ml of 1 M HCl was added to the enriched bicarbonate bottle via a glass pipette passing through the top of the chamber every two days for 45 days to generate ^13^CO_2_ *in situ*. A small fan (d = 8 cm) was turned on for 30 min after the addition of acid to facilitate even distribution of ^13^CO_2_ within the growth chamber.

### Experiment 1. Condition of Crabs

To evaluate relative contributions of organic matter from mangrove leaf litter and sediment to crab’s diet, 18 crabs were randomly divided into three groups, each provided with either only mangrove leaf litter (the L treatment), only sediment (the S treatment), or a combination of both leaf litter and sediment (the L+S treatment). Each crab was allocated to an experimental tank (l×w×h = 40×30×25 cm) such that crab density was equivalent to ca. 9 individual per m^2^. Tanks of the S and L+S treatments were filled with 3 cm of mangrove sediment and those of the L treatment were filled with 3 cm sand. Sand was collected from the foreshore of a local beach, cleared of organic debris by elutriation before adding into the tanks. It is assumed that the low organic matter oceanic sand would serve only as a substrate but not a significant source of carbon for the crabs (organic contents of the sediment and sand used in this experiment were 5.27±0.33% and 0.80±0.02%, respectively). Crabs were maintained at temperature of 24°C and a photoperiod of 16 h light : 8 h dark. A recirculation water supply system was set up for each tank such that seawater of salinity of 25 (PSU) was supplied to each tank according to a semi-diurnal tidal cycle. The reservoir water was replaced with fresh sea water at the end of every week.

Crabs in the L and L+S treatments were provided one *Avicennia* leaf per day while those in the S treatment were not. Uneaten feed was removed from the tanks the following morning. Survival of crabs in the three treatments was recorded every day. After 63 days, all crabs were collected, rinsed with distilled water, and stored at −20°C until dissection of muscle and hepatopancreas tissues from each individual. These tissues and the remaining parts of each crab were freeze-dried and weighed. Hepatosomatic index (HSI) of each crab was determined as the percentage of the dry weight of the hepatopancreas to the rest of the body [32]. Freeze-dried muscle tissues were used for fatty acid analysis.

### Experiment 2. Leaf Litter Assimilation

Another batch of crabs was fed dual ^13^C and ^15^N enriched mangrove leaves for four weeks and temporal changes in stable isotope values of their muscle tissue were studied. Crabs were randomly assigned into the treatment group (32 crabs) and the control group (8 crabs), each were allocated to individual rearing compartments (13×7×4 cm, containing 50 ml of seawater). Crabs in the treatment and control group were provided one enriched and one non-enriched *A. marina* leaf every three days, respectively. Potential stable isotope enrichment in crab tissue due to the utilization of water containing leachate from enriched leaves was assessed by soaking one enriched leaf in each control compartment. This leaf was separated from the crab by two layers of plastic sheets, which had small holes at alternate positions at the bottom, allowing free movement of water across the sheets but preventing the crabs from direct access to the leaf. Enriched leaves in the control compartments were also replaced by fresh ones every three days. Crabs were maintained at temperature of 24°C and a photoperiod of 16 h light: 8 h dark. Water in the rearing compartments was changed once every week.

Eight crabs fed on the enriched-leaf diet were randomly sampled every week for four weeks (these sampling events hereafter are referred to as T1, T2, T3 and T4, respectively). At each sampling time, feed was removed from rearing compartments; crabs were then left to evacuate their gut for 24 h, rinsed with distilled water, and stored at −20°C until dissection. Control crabs were sampled similarly at the end of week 4. Crabs were dissected to collect muscle tissue, which was then dried at 60°C for 24 h. Eight ‘field’ crabs were also dissected to collect samples for T0 sampling event. To calculate assimilation efficiency, faeces were collected twice daily, dried at 60°C for 24 h. Faeces of individual crabs were pooled together at the end of the experiment. Dried muscle tissue and faeces from individual crabs were used for stable isotope analysis.

### Experiment 3. Trophic Discrimination Values of Potential Food Sources

Carbon and nitrogen trophic discrimination values (Δδ^13^C and Δδ^15^N, respectively) in *P. erythodactyla* for three common food sources (mangrove leaf litter, microphytobenthos and animal food) were determined by a third feeding experiment. Crabs were fed freshly senescent *A. marina* leaves, MPB or frozen prawn muscle (*Metapenaeus* spp.) for eight weeks. Previously work on similar animals (S.Y. Lee, unpublished data) suggests that eight weeks is usually sufficient for tissue turnover to result in isotopic equilibrium with the diet. The same feeding experiment was conducted twice (in 2011 and 2012). Twenty crabs were used in the first experiment, half of which was fed prawn while the other half was offered mangrove leaves. In the latter experiment, 40 crabs were randomly assigned into two groups of 20 individuals each, which had either MPB or mangrove leaf diet. Crabs were maintained in individual rearing compartments (13×7×4 cm, containing 50 ml of seawater). Feed was provided *ad libitum*. Uneaten glass filter containing MPB and leaves were replaced with fresh ones the next morning while unconsumed prawns were removed at the end of the day.

At the end of week 1, 3, 5 and 8 (hereafter referred to as T1, T3, T5 and T8, respectively), five crabs were samples from each diet in the MPB-feeding experiment while the numbers of crabs sampled in the prawn-feeding experiment were 1, 2, 2 and 5, respectively. These experimental crabs and five ‘field’ crabs (T0) of each feeding experiments were treated similarly with those sampled in experiment 2.

#### Stable isotope analysis

Carbon and nitrogen stable isotopic values of muscle tissue and faeces of crabs, *Avicennia* leaves, MPB, prawn used as food sources were determined using a Europa GSL (Sercon) elemental analyzer coupled to a Hydra 20–22 (Sercon) isotope ratio mass spectrometer in continuous flow mode. Samples are weighed in tin capsules (about 1 mg for animal sample and 4 mg for MPB and plant samples). PeeDee Belemnite and atmospheric air were used as standards for C and N, respectively. Stable isotope values are reported in *δ*-notation (‰), i.e. δ^13^C or δ^15^N = (R_sample_/R_standard_−1)×1000, where R is C^13^/C^12^ and N^15^/N^14^ ratios for carbon and nitrogen analyses, respectively. The analysis also provided %C and %N of the samples, from which assimilation efficiency (A) was determined by the following equation:
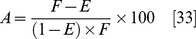
where F = fraction of carbon or nitrogen in feed; and E = fraction of carbon or nitrogen in faeces.

#### Fatty acid analysis

Freeze-dried tissues were ground into powder using a mortar and pestle. Lipid extraction was performed by the one-step method of Abdulkadir and Tsuchiya [34]. In brief, each sample was mixed with 5 ml of hexane and 2 ml of 14% BF_3_ in methanol in a 50 ml glass tube. The tube was heated under reflux on a hot plate at 100°C for 120 min and continuously stirred using a magnetic stirrer. The tube was left to cool to room temperature before 1 ml of hexane and 2 ml of distilled water were added. The tube was then vortexed for 1 min and then centrifuged at 2500 rpm for 3 min. The upper phase was transferred into a fresh tube using a Pasteur pipette. Fatty acid methyl esters (FAMEs) were separated from the extracted FA mix by thin layer chromatography following the method of Meziane and Tsuchiya [35].

FAMEs were then analyzed using a Varian CP-3800 gas chromatograph (GC) with an Omegawax 320 fussed-silica capillary column (Supelco) and flame ionisation detector. Helium was used as carrier gas. Column flow rate was set at 1.5 ml/min. Injector and detector temperatures were set at 240°C and 260°C, respectively. Temperature of the column oven was programmed at 60°C, held for 1 min, then increased to 150°C at the rate of 40°C/min, held for 3 min, then increased to 210°C at 3°C/min, held for 17 min, then increased to 240°C at 5°C/min and held for 10 min. The resulting peaks were identified by comparing their retention times to those of standard FAMEs (Supelco).

Percentage contribution to total FA of FA markers of mangroves (18∶2ω6, 18∶3ω3, and the long-chain FA 26∶0, 28∶0, 30∶0), branched FAs makers of bacteria (15∶0 iso, 15∶0 ant, 16∶0 iso, 16∶0 ant, 17∶0 iso, 17∶0 ant), and MPB (20∶5ω3, a diatom marker) in the crab muscle tissues were determined. These FA markers were selected based on Meziane and Tsuchiya [35] and Hall *et al.* [36], who used FA analysis to investigate the transfer of mangrove organic matter in *P. erythodactyla*.

### Data Analysis

One-way ANOVA (α = 0.05) was used for the comparisons of HSI, the abundance of FA biomarkers, temporal changes in stable isotope values of crabs fed enriched mangrove leaves. Tests for normality (Shapiro-Wilk’s test) and homogeneity of variance (Levene’s test) were performed to check if assumptions of ANOVA were met before analysis. The Kruskal-Wallis H test was used instead of one-way ANOVA when these assumptions were violated. If significant ANOVA or Kruskal-Wallis H test results were obtained, statistical difference between specific treatments were determined by applying Tukey’s Honestly Significant Difference (HSD) post-hoc test or Mann-Whitney U test, respectively.

FA profiles of crabs of the three treatments in the leaf litter utilization study were compared using multivariate analyses with PRIMER version 6 software. Exploratory multi-dimensional scaling (MDS) was conducted based on similarity data built using the Bray-Curtis similarity co-efficient. No transformations were used to avoid giving artificial weight to FAs that only have minor contributions to FA profiles. Statistical differences between treatments were determined using ANOSIM and similarities between them were estimated using the SIMPER function.

#### Determination of trophic discrimination values

Change in isotopic value of the consumers in response to diet shift can be modelled as a function of time as
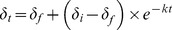
where δ_t_ is the isotopic value of the tissue at time t (in days); δ_f_ is the isotopic value when consumer reaches isotopic equilibrium with the new diet; δ_i_ denotes isotopic composition before the diet shift; and k is the turnover rate of the isotope of interest in the tested tissue [37]. This exponential model was fit to the C and N isotopic data of each feeding experiment to determine the isotopic composition of the muscle at equilibrium with the tested diet (δ_f_ ) by least square method using SigmaPlot 10.0. Model fitting was performed with the δ_i_ term was fixed to the mean isotopic values of the T0 samples. Food specific trophic discrimination factors (Δδ^13^C or Δδ^15^N) were then calculated as the difference between δ_f_ and the mean isotopic value of the food.

#### Assessing diet composition by the IsoConc mixing model

Using the trophic discrimination values determined from experiment 3, the stable isotope values and the C and N concentrations of the tested food, the contribution of these food sources to the crabs’ diet was assessed using the IsoConc mixing model [38]. To compare the diet compositions predicted based on our determined isotopic discrimination values with those estimated based on the trophic fractionation factors that are commonly used in food web data analysis, IsoConc mixing model was also run using the mean trophic discrimination values reported for aquatic consumers of 0.5‰ for δ^13^C and 2.9‰ for δ^15^N [39] with the same data set for other parameters.

## Results

### Experiment 1. Condition of Crabs

**Figure 1:**
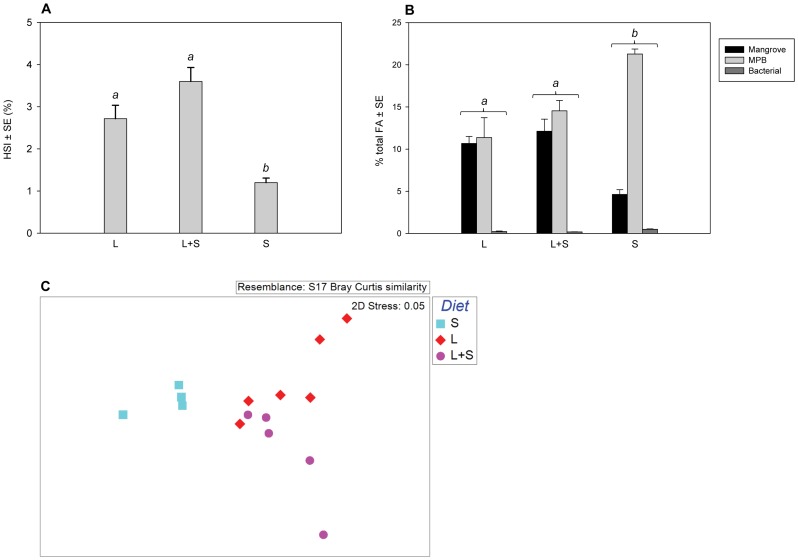
Influence of available organic matter on the fitness of *P. erythodactyla*. Crabs were offered mangrove leaves only (L, n = 6), mangrove leaves and sediment (L+S, n = 5) or sediment only (S, n = 4) for 63 days. Means HSI (A) and % to total FA in muscle tissue of FA biomarkers of the mangrove leaf litter, bacteria and MPB (B) were compared by one-way ANOVA or Kruskal-Wallis H non-parametric tests. Means marked with different letters are significantly different (Tukey’s HSD or Mann-Whitney U, p<0.05). Similarity of FA profiles of crabs in the three treatments was compared by ANOSIM and graphically presented in a MDS plot (C).

All six crabs on the sole mangrove leaf diet (the L treatment) survived through the nine-week experiment. Number of survivors in the L+S treatment, in which crabs had access to both mangrove leave and organic matter from the sediment, and the S treatment, where sediment was the only food source, were five and four, respectively. The type of available food sources significantly affected the hepatosomatic index of the crabs (one-way ANOVA, p = 0.001). Post-hoc analysis showed that there was no significant difference between the L and L+S crabs (Tukey’s HSD, p = 0.116). HSI of the S crabs was, however, significantly lower than those of both the L (p<0.001) and L+S treatments (p = 0.009) (Fig. 1A).

Fatty acid analysis showed that there were significant differences in the proportions of mangrove (one-way ANOVA, p = 0.001), bacterial (one-way ANOVA, p = 0.002), and MPB (Kruskal-Wallis H, p = 0.014) FA biomarkers to total FA in crab muscle tissue between treatments. Proportions of all three FA biomarkers were not significantly different in crabs which had access to mangrove leaves in the presence (the L+S treatment) or absence (the L treatment) of additional organic matter from the sediment (Tukey’s HSD post-hoc test, p = 0.58 for mangrove, p = 0.98 for bacterial, and Mann-Whitney U test, p = 0.46 for MPB markers). Crabs in the S treatment had significantly lower percentage of mangrove FA biomarker but significantly higher proportions of MPB and bacterial biomarkers (p<0.05) than those offered mangrove leaves (Fig. 1B).

A similar trend was also obtained when the fatty acid profiles (i.e. the collections of all FA detected from the muscle tissue) of crabs in the three treatments were compared by ANOSIM analysis. There were no significant differences in the profiles of the L and the L+S crabs (p = 0.08). The FA profiles of crabs of the S treatment, however, were significantly different from those of the L and L+S treatments (p≤0.001). In addition, SIMPER analysis indicated that similarity between the S treatment and the other two treatments (<80% similarity) was lower than that between the L and L+S treatments (85% similarity). This confirms the observed separation of the S treatment from the L and L+S treatments on the MDS plot (Fig. 1C).

### Experiment 2. Leaf Litter Assimilation

**Figure 2:**
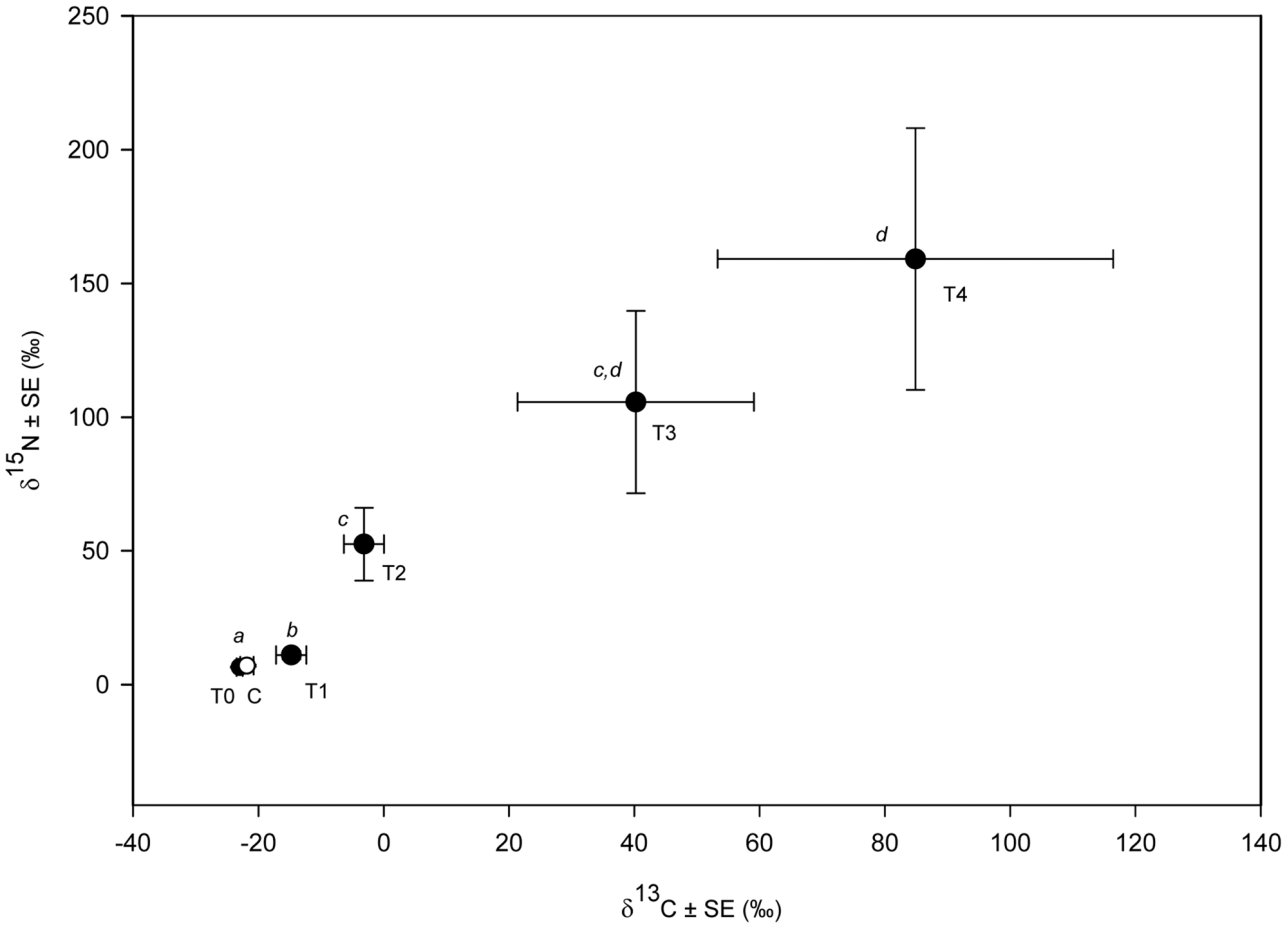
Temporal changes in δ^13^C and δ^15^N values of *P. erythodactyla* fed enriched mangrove leaves. Shown in the plot are the mean δ^13^C and δ^15^N values of crabs sampled at the start of the feeding experiment (T0, n = 7), at the end of every week (T1–T4, n = 8 for each sampling event), and those in the control (C, open circle, n = 8). Data points marked with different italic letters are significantly different (Mann-Whitney U tests, p<0.05).

Muscle tissues of crabs fed the enriched mangrove leaves were clearly enriched in both ^13^C and ^15^N than those of the field crabs. After only one week on the enriched-leaf diet (T1), stable isotope values of the crabs were significantly higher than those of the field crabs (T0, Mann-Whitney U tests, p = 0.006 for δ^13^C and p = 0.009 for δ^15^N). The T4 crabs were significantly enriched in both ^13^C and ^15^N than crabs of the T1 and T2 sampling events but not significantly different from the T3 crabs (Fig. 2), showing that the enrichment was slowing down by the end of the feeding experiment. Mean δ^13^C and δ^15^N of the field crabs and those in the control treatment, in which crabs were provided non-enriched leaves but exposed to water containing leachate from enriched ones, were not significantly different (p>0.05). Enrichment in crabs fed on the enriched leaves is, therefore, solely attributed to the digestion and assimilation of the mangrove leaves, with negligible contamination from the leachate. Crabs assimilated C and N from the mangrove leaves with an efficiency of 36.0 and 56.6%, respectively.

### Experiment 3. Trophic Discrimination Values of the Potential Food Sources

**Figure 3:**
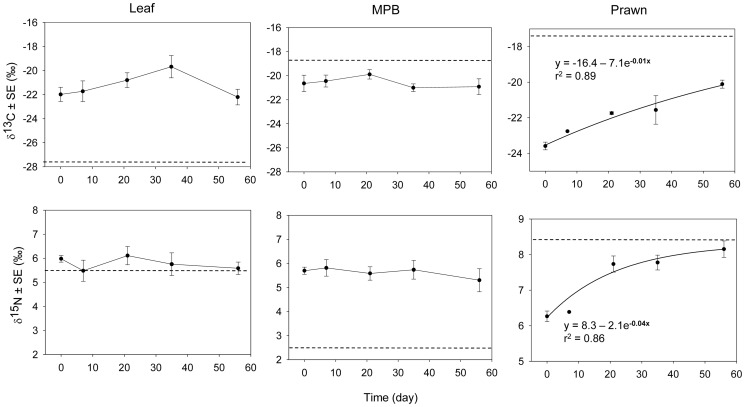
δ^13^C and δ^15^N profiles of crabs on the mangrove leaf, MPB, and prawn diet. Temporal changes in stable isotope values of the muscle tissues of crabs fed the potential dietary items are indicated by the solid lines. Mean δ^13^C and δ^15^N values of the tested food are presented as the broken horizontal lines. For the prawn-feeding data, the best fitting curves are plotted with the respective exponential functions and r^2^ values.

There are no obvious temporal changes in either δ^13^C or δ^15^N in crabs fed mangrove leaves and MPB (One-way ANOVA, p>0.05, Fig. 3). The exponential equation predicted isotopic compositions of the consumer in response to diet shift, hence, was not fitted to these data. C and N isotopic profiles of crabs on the prawn-diet, however, showed a good fit with the model (p<0.001, r^2^ = 0.89 and 0.86, respectively). δ_f_ values were estimated to be −16.4‰ (p = 0.037) and +8.3‰ (p<0.01) for δ^13^C and δ^15^N, respectively.

Table 1 presents the trophic discrimination values (Δδ^13^C and Δδ^15^N) determined for each food item. Δδ^13^C and Δδ^15^N for the prawn diet were calculated from the differences between the respective δ_f_ values estimated from model fitting and the mean stable isotope values of the prawn muscle tissue. For the mangrove leaves and MPB, trophic discrimination factors were determined as the differences between the mean stable isotope values of the final sampling event and those of the food (this calculation will be justified in the discussion).

**Table 1:** Data used in the estimation of relative contributions of mangrove leaf, MPB and animal tissue in the diet of *P. erythodactyla* using the IsoConc mixing model.

Food	δ^13^C (‰)	δ^15^N (‰)	Δ δ^13^C_ (Crab-Food)_ #	Δ δ^15^N_ (Crab-Food)_ #	[C] (%)	[N] (%)
Mangrove	−27.7	+5.5	+5.5	+0.1	45.6	0.8
MPB	−19.0	+2.3	−1.9	+3.0	30.6	5.4
Prawn	−17.5	+8.3	+1.1	0.0	41.1	12.3

# Δ δ^13^C_(Crab-Food)_ and Δδ^15^N_(Crab-Food)_ were determined by subtracting the mean C and N isotopic values of the food from the asymptotic values of the exponential curves for the prawn-diet data or the mean isotopic values of the last time point samples for the leaf and MPB diet data (Fig. 3, refer to text for justification).

### Assessing Diet Composition by the IsoConc Mixing Model

**Figure 4:**
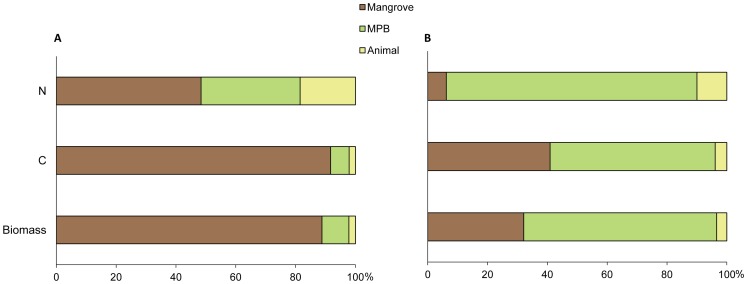
Estimation of the diet composition of *P. erythodactyla* by the IsoConc mixing model. Percentage contribution of the mangrove leaf, MPB and animal food sources in the crab’s diet were estimated by the IsoConc mixing model using (A) our determined isotopic discrimination values (Table 1) and (B) the global means of trophic discrimination values for aquatic food web of 0.5‰ for C and 2.9‰ for N [39].

Results of IsoConc modelling using our trophic discrimination values (Table 1) show that mangrove leaf is the primary contributor to the diet of *P. erythodactyla* in terms of biomass (89%) and C (92%) but less important in term of N (48%, Fig. 4A). On the contrary, animal tissue makes up a minor proportion of the biomass (2%) and C intake (2%) but has a significant contribution to N (19%). Modelling based on the global mean trophic fractionation values reported for aquatic consumers ([39]), however, suggests that MPB but not the mangrove leaf would play a key role in the nutrition of this crab. In this scenario, mangrove litter was estimated to account for only 32, 41 and 6% of the biomass, C and N intakes, respectively (Fig. 4B).

## Discussion

### The Trophic Role of Mangrove Leaf Litter

Results of experiment 1 suggest that mangrove leaf litter was the ‘staple’ food of *P. erythodactyla* while the sediment organic matter sources have a minor contribution to its diet. Crabs on the mangrove litter only diet had comparable fitness and fatty acid profiles to those that had access to additional food sources available from the sediment, such as benthic bacteria, micro-algae or aged detritus. Feeding on the sediment alone, on the contrary, may reduce the fitness of the crabs as indicated by the significant lower HSI (a proxy for fitness) of crabs in this treatment (Fig. 1). We were able to maintain *P. erythodactyla* for more than six months on the sole mangrove leaf diet in the laboratory with a good number of them moulted during that time (unpublished data), suggesting that these crabs had access to sufficient food supply [40]. Bacteria and benthic microalgae in the sediment may provide some subsidy to the N deficit leaf diet, but the crabs are not likely to depend strongly on this source as has been suggested from some studies [9], [28]. From the analysis of C and N balance in the grapsid crab *Neoepisesarma versicolour*, Thongtham and Kristensen [41] also reported that mangrove C was the major C source of this crab while sediment bacteria and MPB, due to their low availability, may have only a minor contribution to the crab’s N requirement. In addition, leaf tissues were the dominant food items found in the gut of other mangrove grapsids such as *Chiromanthes onychophorum* [22], *N. versicolour*, *N. mederi* [*23*], *Aratus pisonii* [21].

Our feeding experiment using δ^13^C and δ^15^N enriched leaf (Fig. 2) provides direct evidence of the assimilation of mangrove organic matter in *P. erythodactyla.* In previous studies on the feeding ecology of mangrove grapsids, the contribution of mangrove leaf litter in their diets has been demonstrated from observations of the crabs’ foraging activities on leaf litter [19], [42], the presence of leaf tissues in the stomach contents [22]–[23], the growth and survival of crabs in long-term feeding experiments [43], and the transfer of terrestrial plant biomarkers such as long-chain fatty acids to the animals [36]. The lack of convincing direct evidence showing the assimilation of mangrove organic matter in these detritivores is obviously one of the reasons why the role of mangrove leaf litter in their diets has been questioned in some recent studies [9], [28]. We also showed that *P. erythodactyla* assimilated C and N from *A. marina* at efficiencies (36% for C and 57% for N) comparable to those reported for *Neosarmatium smithi* fed on *Ceriops tagal* leaf detritus [44] and *N. versicolour* consuming brown (for % C assimilation) and green (for % N assimilation) leaves of *Rhizophora apiculata* [41].

### The Change of Isotope Compositions in Experiment 3

When a new diet is introduced, isotopic compositions of the consumer will shift gradually toward those of the diet in time following an exponential function, i.e. 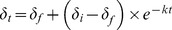 [37], [45]–[46]. The isotope values of crabs on the prawn-diet showed a similar trend (Fig. 3) and fitted well to the proposed exponential model. Asymptotic values of the exponential curves (i.e. the δ_f_ term of the exponential function) were, therefore, considered as the expected isotope values of the crab muscle tissue when it reached equilibrium with the prawn food, which was subsequently used in the calculation of trophic discrimination values (Table 1). It must be noted that there was only a small difference between δ_f_^15^N (+8.3‰) and the mean δ^15^N (+8.2‰) of the samples collected at the final sampling event (T8), while the difference in δ^13^C was larger, −16.4 vs. −20.1‰, respectively. This suggests that δ^15^N of the muscle had almost approached equilibrium with the diet within the time frame of the experiment but C isotope composition may require a longer feeding time to reach isotopic equilibrium. A similar response resulting from diet shift was reported in the mysid *Mysis mixta*, whose δ^15^N reached equilibrium with the *Artemia* used as feed about 8–9 weeks after the diet switch while δ^13^C took longer than 12 weeks to attain isotopic equilibrium [47].

In contrast to the prawn-feeding treatment, isotopic compositions of crabs on the mangrove leaves and MPB diets stayed more or less constant during the experiment (Fig. 3). Herbon and Nordhaus (2013) also reported the lack of change in δ^13^C in the sesarmid crabs *Episesarma singaporense* and *E. versicolor* over the 12 week long feeding experiment, in which *R. apiculata* senescent leave was provided as the sole dietary item. Stabilization of isotopic values in our case may due to: 1) no food ingestion and/or assimilation in the tested animals; 2) the time frame of the experiment was not long enough for an isotopic change following diet switch to occur; 3) the tested diet was similar to the natural diet of the animal; or 4) isotopic discrimination values of the tested diet were coincidentally similar to the difference in stable isotope values of the consumer from the diet.

In our feeding experiments, feed consumption in all experimental crabs was confirmed by the observation of feed removal and defecation. If food was ingested but not assimilated, the animal would have been on long term starvation. Reduction in food consumption and starvation often cause enrichment in δ^15^N of the animals [48]–[51]. The increase in the ^15^N/^14^N ratio is probably due to the same mechanism that causes trophic discrimination of nitrogen: the animals metabolized their own proteins, continuously excreted ^14^N without replenishment from the diet, and thus progressively become enriched in ^15^N [52]. The lack of change in δ^15^N of the MPB and leaf feeding crabs during the feeding experiments suggests that these animals were not starved, thus ruling out the first explanation.

The results of the prawn-feeding treatment in experiment 3 and the enriched-leaf feeding experiment showed significant enrichment of both δ^13^C and δ^15^N after the first three- and one-week periods on the tested diets (Fig. 2 and 3). Eight weeks would therefore be long enough for any change in isotope composition in crabs’ muscle tissue to be detected. Stabilization of stable isotope values throughout the experimental period, therefore, could be attributed to either of the last hypotheses. Under either scenario, difference between the mean stable isotope values of crabs of the last time point from values of the corresponding diet would be close to the true trophic discrimination values for the food of interest (Table 1).

### Trophic Discrimination Values

Our determined trophic discrimination values (Table 1) are significantly different from the mean literature values (e.g.[39], [53]–[54]). These reviews, however, also indicated that variability in Δδ^13^C and Δδ^15^N is significant, i.e. values for specific pairs of consumer-diet vary widely from the global mean values. For example, McCutchan *et al.* [53] showed that C and N discrimination values reported in literature may span the range of −2.7 to +5.5‰ and −2.4 to +9.2‰, respectively. Variation in trophic discrimination values are attributed to food quality (e.g. lipid and protein contents), tissue type analyzed, feeding mode (e.g. fluid-feeding vs. others), and habitat of consumers [55]–[57]. However, mechanisms for the discrepancies are obscure.

Our results suggest that the degree of trophic discrimination is specific to particular food-consumer pair, with a variation of >7‰ for Δδ^13^C and ∼ 3‰ for Δδ^15^N between the three food types tested. Large variations in the C and N isotopic discrimination values of different food sources have also been reported in other animals but smaller differences are more common [39]. For examples, locust *Locusta migratoria* fed with corn and wheat had differences in Δδ^13^C and Δδ^15^N of these dietary items at 5.3‰ and 2.8‰, respectively [58]. In the grasshopper *Melanoplus sanguinipes*, Δδ^15^N of the corn seedling diet was 2.5‰ higher than that of the wheat seedling diet [59]. Indeed, trophic discrimination values may be influenced by food quality. Changes in nitrogen discrimination values in response to variations in protein quality and quantity [60] and C/N ratio [61] of diets have been reported. In addition, selective assimilation and differential routing of nutrient components (i.e. carbohydrates, proteins, lipids), which are expected to differ among the foods tested in our experiment, may also have contributed to the variation in trophic discrimination factors [52], [62].

The difference in C^13^/C^12^ ratio between the mangrove leaf and *P. erythodactyla* (5.5‰) is significantly higher than the commonly assumed trophic discrimination values used for analysing and interpreting grapsid crab food web studies. However, our Δδ^13^C_(crab-mangrove)_ is close to C discrimination values recorded for *R. apiculata* in the grapsid crabs *E. singaporense* (5.1‰) and *E. versicolour* (4.1‰) [63]. Interestingly, our value also only slightly deviates from the mean (5.2) and median (5.6) of the Δδ^13^C values of the grapsid crab-mangrove leaf potential feeding link reported in the literature (Table 2). Caut *et al.* [55] reported a negative linear relationship between Δδ^13^C and food δ^13^C, which is consistent with the large discrimination value we have recorded for the crab-mangrove leaf feeding relationship. The high Δδ^13^C may be due to selective assimilation of ^13^C-enriched dietary components. Carbohydrates such as monosaccharides or cellulose are often more enriched than bulk leaf tissue by ∼1 ‰ [64]–[66]. This difference could increase in extreme growing conditions, e.g. the leaves of *A. marina* growing in a hypersaline lagoon demonstrated a difference up to 5‰ [67]. In leaves of mangroves and other terrestrial plants, δ^13^C values of individual amino acids may vary greatly; variations of *ca.* 20‰ or more have been recorded [68]–[69]. Selective assimilation of carbohydrates or the more enriched amino acids, therefore, would increase the discrimination value. The unusually high Δδ^13^C_crab-mangrove_ values probably reflect some interesting and potentially unique features of the digestive physiology of the mangrove grapsid crabs, which invites further investigation. Nonetheless, these differences from the assumed values used in analyzing stable isotope data, whether through direct comparison or mixing model estimations, have significant implications for the results (see below).

**Table 2:** Difference in δ^13^C values of the mangrove grapsid and their associated mangrove leaf litter from the literature.

Crab species	Mangrove species	Crab δ^13^C	Mangrove δ^13^C	Δ δ^13^C	Reference
*Australoplax tridentata*	AM	−19.2	−27.9	8.7	[80]
	AM	−2.8	−28.8	6	[10]
*Episesarma tetragonum*	AO	−24.2	−28.6	4.4	[29]
	AO/BG/RA/RM	−25.2	−30.4	5.2	[81]
*Episesarma versicolor*	AO	−23.9	−28.6	4.7	[29]
	AM/AO/EA	−25.4	−28.6	3.2	[81]
*Helice formosensis*	KC	−21.5	−28.3	6.8	[82]
*Helograpsus haswellianus*	AM	−21.4	−27	5.6	[9]
*Neoepisesarma versicolor*	RA	−24.3	−28.5	4.2	[27]
	RM	−24.2	−29.3	5.1	[9]
*Paragrapsus laevis*	AM	−21	−27.8	6.8	[9]
*Parasesarma asperum*	AO	−25.5	−28.6	3.1	[29]
	AM/AO/EA	−23.8	−28.6	4.8	[81]
*Parasesarma erythodactyla*	AM	−22	−27.8	5.8	[9]
	AM	−22	−27.9	5.9	[80]
	AM	−23	−28.8	5.8	[10]
	AM	−20.7	−27.8	7.1	[83]
*Parasesarma plicata*	KC/AM/AC	−24	−26.2	2.2	[84]
*Parasesarma plicatum*	AM/AO/EA	−19.5	−28.6	9.1	[81]
*Perisesarma bengalensis*	AM/AO/EA	−25.4	−28.6	3.2	[81]
	RA/EA/BG	−25.7	−31.4	5.7	[81]
*Perisesarma bidens*	KC/AM/AC	−24.2	−26.2	2	[84]
*Perisesarma dussumieri*	AO/BG/RA/RM	−27.3	−30.4	3.1	[81]
	RA/EA/BG	−25.8	−31.4	5.6	[81]
*Perisesarma guttatum*	AM/CT/XG/RM	−23.3	−29.3	6	[81]
*Perisesarma* sp.	AM/CT/XG/RM	−22.1	−29.3	7.2	[81]
*Psuedosesarma crassimanum*	AO/BG/RA/RM	−24.3	−30.4	6.1	[81]
*Sesarma* spp.	AA	−24.0	−27.4	3.4	[14]
Mean		−**23.1**	−**28.3**	**5.2**	
SD		**2.6**	**2.2**	**1.8**	
Median		−**23.9**	−**28.6**	**5.6**	

AM – *Avicennia marina*; AO – *A. officinalis*; AC – *Aegiceras corniculatum*; BG – *Bruguiera gymnorhiza*; CT – *Ceriops tagal*; EA – *Excoecaria agallocha*; KC – *Kandelia candel*; RA – *Rhizophora apiculata*; RM – *Rhizophora mucronata*; XG – *Xylocarpus granatum*.

### Implication of the Food-Specific Trophic Discrimination Values for the Mangrove-Grapsid Link

Mixing model calculations using our food-specific isotopic discrimination values suggest a significantly higher contribution of mangrove leaf litter to the crab’s diet compared to results generated using the global mean values reported for aquatic consumers [39]. Increase in the contribution from mangrove leaf litter also means that contribution from the other common sources, such as the MPB, would decrease. The diet composition predicted using the experimentally determined values is in line with reports on the dominant proportion of leaf tissues in the gut contents of the mangrove grapsids. Microalgae and animal tissue remnants were also found, but at much lower fractions [21]–[23]. Our data question the proposed dominant dietary role of MPB based on modelling results obtained using the global mean trophic discrimination values. Firstly, there is no direct evidence on the consumption of large amount of MPB in mangrove grapsids. In addition, although MPB does offer some apparent nutritional advantages, such as a higher N content and presumably higher digestibility, the grapsid crabs’ feeding appendages are not morphologically adapted to collecting microscopic food from the sediment. Mangrove grapsids have chelae with pointed ‘finger-tips’ that are more adapted to capturing, tearing and cutting large food items, rather than the spoon-tip feeding chelae found in deposit-feeding species such as the fiddler crabs (*Uca* spp.). Intertidal crabs that rely on microscopic food (e.g. MPB or the meiofauna) also possess specialised mouth parts or sediment processing behaviour, which are not present in the grapsids, to help efficiently extract food particles from the large volume of sediment that needs to be handled, e.g. ‘floatation feeding’ in soldier crab *Mictyris longicarpus* [70]. Further, the limited growth of microalgae on the poorly lighted mangrove forest floor [71]–[72] would hardly meet nutritional requirement of the highly abundant grapsid communities if it is their main food item. The strong competition for MPB from ocypodid crabs, such as *Heloecius cordiformis* on sub-tropical eastern Australian coast, which are also found at high density in more open mangroves, further reduces the availability of MPB to the grapsids. From the analysis of the N budget of the grapsid crab *N. versicolor* and the estimation of the nitrogen content of MPB in the sediment at the same site, Thongtham and Kristensen [41] showed that this crab must consume an unrealistically high amount of sediment to obtain sufficient N for its growth. N demand in the mangrove grapsids was more likely met by occasional consumption of animal tissue, through cannibalism, predation of other invertebrates or scavenging rather than regular consumption of MPB [21], [27].

Our results not only confirm the significance of grapsid crabs in directly processing mangrove leaf organic matter, but also highlights the risk of applying ‘global’ trophic discrimination values to analyzing stable isotope food web data. The values commonly used are mean values derived from a large number of consumer-food combinations, which expectedly result in a wide distribution of actual discrimination values (see [39], [53]–[54]). The mean values, while offering some statistical information on the discrimination values, are of little direct value in analyzing specific feeding relationships [56]. Values specific to potential consumer-food combinations need to be obtained before their application to mixing model calculations. While this may not be logistically feasible for all potential food items, a more practical approach is to obtain the values at least at the food category level (e.g. vascular plant vs. animal food) in animals that use diverse food sources.

Studies on C dynamics in estuaries have been dominated by the ‘outwelling’ paradigm for decades [5], [7], [73]. While earlier rates of export may have been over-estimated [8], recent attention on tropical mangroves has taken a dramatic turn in highlighting the role of mangroves as prime ‘blue-carbon’ storages [74]–[76]. In-situ consumption of fresh mangrove leaf litter by detritivores such as grapsid crabs and gastropods is a major fate of mangrove production, especially in the Indo-west-Pacific [20], [77]. In poorly flushed mangroves with little tidal export, the sediment carbon accumulation rate depends on the balance between litter production and mineralization (in-situ consumption by macro-detritivores and microbial decomposition). Under-estimating detritus utilization resulting from inappropriate use of global trophic discrimination values in isotopic analyses would emphasize the storage role of mangroves while undervaluing the trophic contribution of wetland vascular plant production to coastal food chains. With recent reports on common occurrence of cellulases in estuarine invertebrates [78]–[79], these data together demand a re-examination of the general significance of the detritus-based food chain.

In conclusion, our study confirms the dominant role of mangrove leaf litter in the diet of grapsid crabs. Mechanisms by which these animals can benefit from a diet dominated by an apparently low-quality food item require further investigation. With a capacity to convert the low-quality mangrove C into biomass, the grapsid crabs would mediate the transfer of mangrove primary production to nearshore consumers. This link is particularly important in the Indo-west-Pacific mangroves, where the abundance and diversity of mangrove grapsids are maximal [20]. Other trophic links originating from vascular plant detritus may similarly have been undervalued because of the use of inappropriate trophic discrimination values in past isotopic studies. Our data suggest that the tide has not yet turned for mangrove trophic support to nearshore consumer communities.
